# Association between ethnicity and health knowledge among the floating population in China

**DOI:** 10.1186/s12962-022-00349-0

**Published:** 2022-04-02

**Authors:** Bingxue Han, Hongyi Guan, Ming Guan

**Affiliations:** 1 Xuchang Urban Water Pollution Control and Ecological Restoration Engineering Technology Research Center, Xuchang University, Xuchang, China; 2 College of Urban and Environmental Sciences, Xuchang University, Xuchang, China; 3 Grade 6 Class 7, Xuchang Municipal Xingye Road Primary School, Xuchang, Henan China; 4 Family Issues Center, Xuchang University, Xuchang, Henan China; 5 International Issues Center, Xuchang University, Xuchang, Henan China; 6 School of Business, Xuchang University, Xuchang, Henan China

**Keywords:** Health knowledge categories, Health knowledge sources, Floating population, Ethnic minority, Han majority

## Abstract

**Background:**

Health equity remains a priority concerns by central government in China. This study aimed to explore ethnic gaps in access to health knowledge categories and sources based on the survey data from a publicly available dataset.

**Methods:**

Data were from 2015 China Migrants Dynamic Survey issued by The National Health Commission in China. Descriptive analyses were performed to reflect geodemographic differences in the floating population of ethnic minority (EMFP) and Han majority (HMFP) with Chi-square test. Ethnic gaps in access to health knowledge categories and sources were explored with Poisson regressions, logistic regressions, and bivariate ordered probit regressions.

**Results:**

In the sample, most of participants had inadequate health information literacy. There were significant differences regarding geodemographic factors between EMFP and HMFP. Illiterate EMFP had likelihood to obtain less health knowledge categories (IRR = 0.80, 95% CI 0.77–0.84) and sources (IRR = 0.83, 95% CI 0.80–0.86) as compared to illiterate HMFP. Most of correlations between health knowledge categories and sources were weak in the samples of EMFP and HMFP.

**Conclusion:**

Ethnic disparities in access to health knowledge categories and sources among the floating population in China were confirmed. Further effective efforts should be provided to reduce ethnic disparities in access to health knowledge under the ethnicity-orientated support of public health resource.

## Background

Since the 1950s, China has adopted ethnic policy with official goal of “equality de facto” and given ethnic minorities more political status, which has improved their socioeconomic development [1]. However, ethnic disparities in disease prevalence and control management in China have been documented [2–8] in ethnic minority regions in China since the 2000s. Being one of the basic social demands, ethnic differences in access to health knowledge draw increasing attention in China in recent years. This study was performed to gain a better understanding of ethnic access to health knowledge among the floating population in China.

During the first decade of the twenty-first century, China’s population has been characterized as geographic diversification of destinations for the interprovincial floating population in China’s western and interior regions [9]. The classic theories of population migration are found inappropriate for understanding existence of floating population [10]. The floating population is characterized by low household healthcare expenditure [11], low treatment rate in medical institutions [12], a certain percentage of self-treatment behavior [13], and poor awareness of reproductive health [14] in China. A systematic review reported unhealthy lifestyle and deficient hypertension disease management among floating population in China [15]. Meanwhile, part of China’s floating population might be at increased risk of acquiring the HIV transmission virus [16].

Furthermore, a study with the data of 1% national population sample survey 2005 reported about 70% ethnic minority floating population (EMFP) were from minority autonomous region, three fourth of whom come from rural areas [17]. Currently, EMFP was mostly young adults with deficient education, low incomes, and lack of social security [18] and low wages [19, 20]. Accordingly, the previous studies indicate that there are geodemographic differences between Han and ethnic minority groups in China.

In practice, health inequity among EMFP was neglected [21]. Prior research documented ethnic disparities in infant feeding practices [22] and prevalence of smoking [23]. Simultaneously, a study indicated obvious ethnic disparities in self-rated health were mainly affected by socioeconomic factors [24]. Another study indicates that health information might help mitigate the mental health issues Chinese youth experienced [25]. Similarly, a study in a rural Yunnan, China indicated that health education was shown to be effective in promoting food-health-related knowledge in a rural ethnic minority community [26]. Thus, this study focused on the geodemographic factors associating with health knowledge obtainment. Moreover, it needs to confirm the relationships between health knowledge categories and sources among EMFP in China.

The motivation for this study is a concern that EMFP in China has differential access to health knowledge than Han majority floating population (HMFP). The second part reviewed current relevant literature and designed hypotheses. The third part expounded data source, sampling methods, main variables, and statistical strategies. The forth part explored ethnic disparities in access to health knowledge categories and sources between EMFP and HMFP. The final part discussed the statistical results and concluded the paper.

## Literature review

Health education is acknowledged as an integral component of disease control and can potentially mitigate adverse health outcomes for individuals. There was gender difference with respect to relatively low oral health knowledge among the university students [27]. Furthermore, there was importance difference in health knowledge categories [28]. Remarkably, lack of knowledge was found to affect health/healthcare service utilization [29].

Clinically, a study indicated that orthodontic patients’ awareness of their periodontal health was affected by age, attitude and duration of orthodontic treatment [30]. Regarding common persons, sociodemographic factors could affect health knowledge of the elderly in the sample areas of rural China [31]. Factors affecting women’s knowledge of lifestyle-related risk factors during pregnancy were specifically associated with socioeconomic status [32]. Even more important, the socio-economic and educational factors were the factors that determined a greater level of general knowledge on oral health from the pregnant women [33].

However, a systematic scoping review indicated unavailable health knowledge was reported in the literature [34]. Studies documented lack of requisite health knowledge in clinicians [35], Nepal diabetes patients [36], paediatricians [37], Australian pharmacists [38], health professionals [39], primary care physicians [40], and nurses working in heart failure units [41]. Even worse, the gaps in oral health knowledge among a sample of pediatricians were reported [42]. Health knowledge deficiency poses a serious threat on the improvement of health conditions among the individuals of interest. Various sources of health knowledge should be used to increase health knowledge categories for them. Thus, we generate the first hypothesis:

**Hypothesis 1:**

There existed ethnic disparities in access to health knowledge categories among the floating population in China.

Early studies highlighted education in the health knowledge dissemination. For example, a study indicated significant positive changes in opinions were observed following exposure to the peer education intervention [43]. Early studies highlighted medical staff in the health knowledge transmission. For example, medical professionals could effectively improve the health knowledge of people at high risk of stroke [44]. In addition, multiple studies indicated that health information could be disseminated by WeChat [45, 46], internet/social media [47, 48], visual messages [49], text books [50], blogs [51], social media [52], iPad-based app [53], short-video app TikTok [54, 55], Sina Weibo [56], booklet [57], mass media [58], and web-based educational film [59]. Consistent with literature, we therefore hypothesized:

**Hypothesis 2:**

There existed ethnic disparities in access to health knowledge sources among the floating population in China.

**Hypothesis 3:**

There existed strong correlations between health knowledge categories and sources among EMFP in China.

## Method

### Data source

This study employed the 2015 China Migrants Dynamic Survey (http://www.chinaldrk.org.cn/) data. The survey employed stratified, multi-stage, scale-oriented Probability Proportionate to Size method and covered approximately 10,000 sample points. There were 35 provincial-level units in China including 23 provinces, 5 autonomous regions, 4 municipalities directly under the Central Government, 2 special administrative regions (https://baike.so.com/doc/23635518-24188839.html) and Xinjiang Production and Construction Corps. But, in this study, only 32 provincial-level units were surveyed excluding Taiwan, Hong Kong, and Macau. In the sample, EMFP included Mongolia ethnicity, Man ethnicity, Hui ethnicity, Zang ethnicity, Zhuang ethnicity, Uygur ethnicity, Miao ethnicity, Yi ethnicity, Tujia ethnicity, Buyi ethnicity, Dong ethnicity, Yao ethnicity, Korean ethnicity, Bai ethnicity, Hani ethnicity, Li ethnicity, Kazakh ethnicity, Dai ethnicity, and other ethnicity.

### Main variables

The main socioeconomic variables included gender (female = 0, male = 1), education level (illiteracy = 0, primary school, junior high school, high school/technical secondary school, college, undergraduate, and graduate students = 1), ethnicity (Han majority = 0, ethnic minority = 1), hukou (other = 0, agricultural = 1), first marriage (no = 0, yes = 1), and interprovincial floating (no = 0, yes = 1). The continuous age was coarsely categorized into young group (< 30), middle group (30–60), and old group (> 60). The main regional variables included Pearl River Delta, Yangtze River Delta, Bohai Rim, and other economic zone. Their response options were binary values (no = 0, yes = 1).

Here, health knowledge categories and sources were a series of dummy variables (no = 0, yes = 1). Health knowledge categories included occupational diseases, nutrition, reproduction, chronic diseases, smoking control, mental disorders, tuberculosis, sexually transmitted diseases/acquired immunodeficiency syndrome (STD/AIDS), and other infectious diseases. Health knowledge sources included lecture, books/magazine/CD, radio/TV programmes, face-to-face consultation, online education, community advocacy, bulletin board, and SMS/WeChat.

### Statistical strategies

Descriptive analyses were performed to reflect geodemographic differences between EMFP and HMFP with Chi-square test. Likewise, accessible disparities in health knowledge categories and sources could be exhibited.

To investigate the role of ethnicity in health knowledge gaps between EMFP and HMFP, interaction terms between ethnicity and geodemographic factors were considered as independent variables. Also, health knowledge categories and sources acted as dependent variables. Poisson regressions and logistic regressions with suppressing constant terms were adopted to reflect the associations of ethnicity with health knowledge categories and sources.

Accordingly, Poisson regressions on number of health knowledge categories and sources were conducted to reflect quantitative differences between EMFP and HMFP. Subsequently, logistic regressions were adopted to analyze the associations of interactions between EMFP and geodemographic factors with health knowledge categories and sources.

To reflect a category of specific health knowledge’ reliance on a specific health knowledge source, correlations between health knowledge categories and sources were performed with Stata program bioprobit [60]. The covariates were age (continuous years) and floating years. Three possible correlational results were positive correlation, negative correlation, and no correlation. According to Evans (1996), the strength of the correlation were classified by “very weak” (the absolute value of correlation coefficient (rho): 0.00–0.19), “weak” (the absolute value of rho: 0.20–0.39), “moderate” (the absolute value of rho: 0.40–0.59), “strong” (the absolute value of rho: 0.60–0.79), and “very strong” (the absolute value of rho: 0.80–1.0) [61].

## Results

In this section, the original hypotheses will be statistically explored. In order to reflect ethnic disparities in access to health knowledge categories, ethnic disparities in access to health knowledge sources, and correlations between health knowledge categories and sources, Chi-square test, Poisson regressions, logistic regressions, and bivariate ordered probit regressions were performed. Based on a series of analyses, the original hypotheses would be judged whether or not one of them was accepted.

Totally, mean age of HMFP was 35.579 (± 10.591) years old, while mean age of EMFP was 34.497 (± 11.180) years old. HMFP was averagely older than EMFP. Mean floating time of HMFP was 4.711 (± 4.907) years, while mean floating time of EMFP was 4.757 (± 5.146) years. Thus, mean floating time of HMFP was shorter than that of EMFP.

In Table 1, there were significant differences regarding gender, education level, hukou, first marriage, interprovincial floating, Pearl River Delta, Yangtze River Delta, Bohai Rim, other economic zone, occupational disease, nutrition, reproduction, smoking control, tuberculosis, STD/AIDS, other infectious disease, lecture, radio/TV programmes, face-to-face consultation, online education, community advocacy, bulletin board, and SMS/WeChat between HMFP and EMFP.

**Table 1:** Sample characteristics of floating population by ethnicity

	Column percentage	Han majority (%)	Ethnic minority (%)	Chi-square	P value
Age (N = 206,000)				138.2422	0.000
Young	36.31	33.15	3.16		
Middle	61.31	56.83	4.48		
Old	2.38	2.16	0.22		
Gender (N = 206,000)				20.2281	0.000***
Male	53.07	49.03	4.04		
Female	46.93	43.12	3.81		
Education level (N = 206,000)				2.0e+03	0.000***
No	1.89	1.38	0.51		
Yes	98.11	90.77	7.34		
Hukou (N = 206,000)				87.6745	0.000***
Other	16.41	15.33	1.08		
Agricultural	83.59	76.81	6.78		
First marriage (N = 206,000)				325.7945	0.000***
No	22.59	20.37	2.22		
Yes	77.41	71.78	5.63		
Interprovincial floating (N = 206,000)				1.4e+03	0.000***
No	50.12	45.08	5.04		
Yes	49.88	47.06	2.82		
Pearl river delta (N = 206,000)				20.1735	0.000***
No	92.71	85.36	7.35		
Yes	7.29	6.79	0.50		
Yangtze river delta (N = 206,000)				650.5791	0.000***
No	92.14	76.37	15.77		
Yes	7.86	7.12	0.74		
Bohai Rim (N = 206,000)				726.1827	0.000***
No	92.15	75.89	16.26		
Yes	7.85	7.12	0.73		
Other economic zone (N = 206,000)				1.8e+03	0.000***
No	40.77	38.80	1.97		
Yes	59.23	53.35	5.88		
Knowledge of occupational disease (N = 205,990)				141.4147	0.000***
No	60.24	55.16	5.08		
Yes	39.76	36.98	2.78		
Knowledge of nutrition (N = 205,990)				299.2678	0.000***
No	34.82	31.60	3.22		
Yes	65.18	60.55	4.63		
Knowledge of reproduction (N = 205,990)				69.9840	0.000***
No	33.38	30.53	2.86		
Yes	66.62	61.62	5.00		
Knowledge of chronic disease (N = 205,990)				0.2375	0.626
No	57.98	53.44	4.54		
Yes	42.02	38.70	3.32		
Knowledge of smoking control (N = 205,990)				70.6805	0.000***
No	39.04	35.73	3.31		
Yes	60.96	56.41	4.55		
Knowledge of mental disorders (N = 205,990)				0.7932	0.373
No	80.57	74.26	6.31		
Yes	19.43	17.88	1.55		
Knowledge of tuberculosis (N = 205,990)				382.4506	0.000***
No	62.40	58.06	4.34		
Yes	37.60	34.09	3.52		
Knowledge of STD/AIDS (N = 205,990)				264.1414	0.000***
No	43.67	40.72	2.95		
Yes	56.33	51.42	4.90		
Knowledge of other infectious disease (N = 205,990)				148.4978	0.000***
No	63.03	58.43	4.60		
Yes	36.97	33.72	3.25		
Lecture (N = 189,345)				3.2577	0.071*
No	69.29	64.00	5.29		
Yes	30.71	28.29	2.42		
Books/magazine/CD (N = 189,345)				0.3808	0.537
No	56.77	52.37	4.39		
Yes	43.23	39.92	3.31		
Radio/TV programmes (N = 189,345)				19.2453	0.000***
No	20.26	18.81	1.45		
Yes	79.74	73.48	6.25		
Face-to-face consultation (N = 189,345)				83.8634	0.000***
No	72.64	67.29	5.35		
Yes	27.36	25.00	2.36		
Online education (N = 189,345)				164.7912	0.000***
No	58.09	53.22	4.87		
Yes	41.91	39.07	2.84		
Community advocacy (N = 189,345)				43.9996	0.000***
No	61.51	56.96	4.54		
Yes	38.49	35.33	3.17		
Bulletin board (N = 189,345)				37.0847	0.000***
No	16.55	15.41	1.14		
Yes	83.45	76.88	6.57		
SMS/WeChat (N = 189,345)				55.6273	0.000***
No	43.70	40.11	3.60		
Yes	56.30	52.19	4.11		

* and *** represent 10 and 1%, respectively

Regarding health knowledge categories (n = 205,990), knowledge of reproduction (66.62%) was the most popular health knowledge category for the total sample followed by knowledge of nutrition (65.18%), smoking control (60.96%), STD/AIDS (56.33%), chronic diseases (42.02%), occupational diseases (39.76%), tuberculosis (37.60%), other infectious diseases (36.97%), and mental disorders (19.43%). Knowledge of reproduction (5.00%) were the most popular health knowledge category for the EMFP followed by knowledge of STD/AIDS (4.90%), nutrition (4.63%), smoking control (4.55%), tuberculosis (3.52%), chronic diseases (3.32%), other infectious diseases (3.25%), occupational diseases (2.78%), and mental disorders (1.55%).

Regarding health knowledge sources (n = 189,345), bulletin board (83.45%) was most utilized by the total sample, followed by radio/TV programmes (79.74%), SMS/WeChat (56.30%), books/magazine/CD (43.23%), online education (41.91%), community advocacy (38.49%), lecture (30.71%), and face-to-face consultation (27.36%). In EMFP, bulletin board (6.57%) was most utilized, followed by radio/TV programmes (6.25%), SMS/WeChat (4.11%), books/magazine/CD (3.31%), community advocacy (3.17%), online education (2.84%), lecture (2.42%), and face-to-face consultation (2.36%).

### Association between ethnicity and number of health knowledge categories and sources

In Table 2, EMFP was likely to obtain more health knowledge categories and sources as compared to young HMFP. Illiterate EMFP had likelihood to obtain less health knowledge categories [IRR = 0.80, 95% Confidence Interval (CI) 0.77–0.84] and sources (IRR = 0.83, 95% CI 0.80–0.86) as compared to illiterate HMFP. EMFP with non-agricultural hukou had likelihood to obtain more health knowledge categories (IRR = 1.08, 95% CI 1.05–1.11) and sources (IRR = 1.08, 95% CI 1.06–1.11) as compared to HMFP with non-agricultural hukou. EMFP not interprovincial floating had likelihood to obtain health more knowledge categories (IRR = 1.06, 95% CI 1.04–1.09) as and sources (IRR = 1.02, 95% CI 1.00–1.04) compared to HMFP not interprovincial floating. EMFP without first marriage had likelihood to obtain less health knowledge categories (IRR = 0.97, 95% CI 0.95–0.99) and more health knowledge sources (IRR = 1.03, 95% CI 1.01–1.04) as compared to HMFP without first marriage.

**Table 2:** Poisson regressions on number of health knowledge categories and sources, IRR (95% CI)

	Number of health knowledge categories		Number of health knowledge sources	
	IRR	95% CI	IRR	95% CI
eth#agg				
NO, young	1 [Reference]		1 [Reference]	
NO, middle	0.98***	0.98–0.99	0.95***	0.94–0.95
NO, old	0.83***	0.81–0.85	0.80***	0.79–0.81
Yes, young	2.37***	2.19–2.57	2.58***	2.43–2.73
Yes, middle	2.32***	2.15–2.51	2.47***	2.33–2.62
Yes, old	1.88***	1.69–2.10	2.12***	1.96–2.29
eth#gender				
No, no	1 [Reference]		1 [Reference]	
No, yes	1.00	0.99–1.00	0.99***	0.99–1.00
Yes, no	1.00	0.98–1.02	1.00	0.99–1.02
eth#edu				
No, no	1 [Reference]		1 [Reference]	
No, yes	1.23***	1.20–1.27	1.24***	1.21–1.27
Yes, no	0.80***	0.77–0.84	0.83***	0.80–0.86
eth#huk				
No, no	1 [Reference]		1 [Reference]	
No, yes	0.92***	0.91–0.93	0.93***	0.93–0.94
Yes, no	1.08***	1.05–1.11	1.08***	1.06–1.11
eth#intpv				
No, no	1 [Reference]		1 [Reference]	
No, yes	0.96***	0.96–0.97	0.98***	0.98–0.99
Yes, no	1.06***	1.04–1.09	1.02**	1.00–1.04
eth#mar				
No, no	1 [Reference]		1 [Reference]	
No, yes	1.02***	1.01–1.03	1.00	0.99–1.00
Yes, no	0.97**	0.95–0.99	1.03***	1.01–1.04
eth#zon1				
No, no	1 [Reference]		1 [Reference]	
No, yes	3.89***	3.76–4.01	3.58***	3.49–3.67
Yes, no	1.06**	1.01–1.11	1.13***	1.09–1.18
eth#zon2				
No, no	1 [Reference]		1 [Reference]	
No, yes	3.54***	3.43–3.65	3.51***	3.43–3.60
Yes, no	1.33***	1.26–1.39	1.15***	1.12–1.19
eth#zon3				
No, no	1 [Reference]		1 [Reference]	
No, yes	3.43***	3.32–3.54	3.39***	3.31–3.47
Yes, no	1.33***	1.28–1.38	1.24***	1.20–1.28
eth#zon4				
No, no	1 [Reference]		1 [Reference]	
No, yes	3.94***	3.82–4.07	3.74***	3.65–3.83
N	205,990		189,345	

*eth* ethnicity; *agg* age group; *edu* education level; *huk *hukou; *intpv* interprovincial floating; *mar* first marriage; *zon1* Pearl River Delta; *zon2* Yangtze River Delta; *zon3* Bohai Rim; *zon4* other economic zone

** and *** represent 5 and 1%, respectively

EMFP not living in Pearl River Delta had likelihood to obtain more health knowledge categories (IRR = 1.06, 95% CI 1.01–1.11) and sources (IRR = 1.13, 95% CI 1.09–1.18) as compared to HMFP not living in Pearl River Delta. EMFP not living in Yangtze River Delta had likelihood to obtain more health knowledge categories (IRR = 1.33, 95% CI 1.26–1.39) and sources (IRR = 1.15, 95% CI 1.12–1.19) as compared to HMFP not living in Yangtze River Delta. EMFP not living in Bohai Rim had likelihood to obtain more health knowledge categories (IRR = 1.33, 95% CI 1.28–1.38) and sources (IRR = 1.24, 95% CI 1.20–1.28) as compared to HMFP not living in Bohai Rim.

### Association between ethnicity and health knowledge categories

In Table 3, old EMFP was less likely to acquire knowledge of occupational diseases (aOR = 0.40, 95% CI 0.28–0.55), reproduction (aOR = 0.23, 95% CI 0.17–0.32), chronic diseases (aOR = 0.22, 95% CI 0.17–0.30), smoking control (aOR = 0.55, 95% CI 0.41–0.75), mental disorders (aOR = 0.05, 95% CI 0.04–0.08), tuberculosis (aOR = 0.05, 95% CI 0.03–0.06), STD/AIDS (aOR = 0.06, 95% CI 0.05–0.08), and other infectious diseases (aOR = 0.13, 95% CI 0.09–0.17) as compared to young HMFP. Female EMFP was less likely to acquire knowledge of occupational diseases (aOR = 0.81, 95% CI 0.76–0.87) and smoking control (aOR = 0.63, 95% CI 0.59–0.67) and more likely to acquire knowledge of nutrition (aOR = 1.17, 95% CI 1.10–1.25) and reproduction (aOR = 1.58, 95% CI 1.48–1.70) as compared to female HMFP.

**Table 3:** Logistic regression on health knowledge categories (N = 205,990), Odds Ratio [95% Confidence Interval]

	Occupational disease	Nutrition	Reproduction	Chronic disease	Smoking control	Mental disorders	Tuberculosis	STD/AIDS	Other infectious disease
eth#agg									
NO, young	1 [Reference]	1 [Reference]	1 [Reference]	1 [Reference]	1 [Reference]	1 [Reference]	1 [Reference]	1 [Reference]	1 [Reference]
NO, middle	0.93^c^ (0.91–0.95)	0.93^c^ (0.90–0.95)	0.80^c^ (0.78–0.82)	1.12^c^ (1.09–1.14)	0.99 (0.96–1.01)	0.97^b^ (0.94–0.99)	1.07^c^ (1.05–1.10)	0.90^c^ (0.88–0.92)	0.99 (0.97–1.02)
NO, old	0.44^c^ (0.41–0.48)	1.03 (0.97–1.10)	0.18^c^ (0.17–0.19)	1.60^c^ (1.51–1.71)	0.83^c^ (0.78–0.89)	0.84^c^ (0.78–0.91)	1.06^a^ (0.99–1.13)	0.39^c^ (0.37–0.42)	0.90^c^ (0.84–0.96)
Yes, young	0.92 (0.72–1.17)	1.18 (0.93–1.49)	0.92 (0.72–1.18)	0.17^c^ (0.13–0.21)	0.89 (0.70–1.13)	0.06^c^ (0.05–0.09)	0.05^c^ (0.04–0.06)	0.15^c^ (0.12–0.19)	0.15^c^ (0.12–0.19)
Yes, middle	0.81^a^ (0.64–1.03)	1.03 (0.82–1.29)	0.79^a^ (0.62–1.01)	0.17^c^ (0.13–0.22)	0.86 (0.68–1.08)	0.06^c^ (0.05–0.09)	0.05^c^ (0.04–0.07)	0.14^c^ (0.11–0.18)	0.15^c^ (0.12–0.20)
Yes, old	0.40^c^ (0.28–0.55)	0.82 (0.61–1.10)	0.23^c^ (0.17–0.32)	0.22^c^ (0.17–0.30)	0.55^c^ (0.41–0.75)	0.05^c^ (0.04–0.08)	0.05^c^ (0.03–0.06)	0.06^c^ (0.05–0.08)	0.13^c^ (0.09–0.17)
eth#gender									
No, no	1 [Reference]	1 [Reference]	1 [Reference]	1 [Reference]	1 [Reference]	1 [Reference]	1 [Reference]	1 [Reference]	1 [Reference]
No, yes	1.24^c^ (1.22–1.27)	0.78^c^ (0.77–0.80)	0.57^c^ (0.56–0.58)	1.00 (0.98–1.02)	1.76^c^ (1.73–1.80)	0.96^c^ (0.94–0.98)	1.01 (0.99–1.03)	0.96^c^ (0.94–0.98)	0.96^c^ (0.95–0.98)
Yes, no	0.81^c^ (0.76–0.87)	1.17^c^ (1.10–1.25)	1.58^c^ (1.48–1.70)	0.99 (0.93–1.06)	0.63^c^ (0.59–0.67)	1.05 (0.97–1.14)	0.98 (0.92–1.04)	1.02 (0.96–1.10)	1.03 (0.97–1.10)
eth#edu									
No, no	1 [Reference]	1 [Reference]	1 [Reference]	1 [Reference]	1 [Reference]	1 [Reference]	1 [Reference]	1 [Reference]	1 [Reference]
No, yes	1.64^c^ (1.50–1.79)	1.69^c^ (1.57–1.82)	1.79^c^ (1.65–1.94)	1.36^c^ (1.25–1.47)	1.42^c^ (1.31–1.53)	1.35^c^ (1.21–1.50)	1.26^c^ (1.16–1.36)	1.59^c^ (1.47–1.72)	1.34^c^ (1.23–1.45)
Yes, no	0.53^c^ (0.46–0.62)	0.60^c^ (0.53–0.69)	0.60^c^ (0.53–0.69)	0.61^c^ (0.53–0.70)	0.61^c^ (0.53–0.69)	0.72^c^ (0.60–0.86)	0.86^b^ (0.76–0.98)	0.61^c^ (0.53–0.69)	0.77^c^ (0.67–0.88)
eth#huk									
No, no	1 [Reference]	1 [Reference]	1 [Reference]	1 [Reference]	1 [Reference]	1 [Reference]	1 [Reference]	1 [Reference]	1 [Reference]
No, yes	0.75^c^ (0.73–0.77)	0.77^c^ (0.75–0.79)	0.92^c^ (0.90–0.95)	0.79^c^ (0.77–0.81)	0.87^c^ (0.85–0.89)	0.77^c^ (0.75–0.80)	0.87^c^ (0.85–0.89)	0.89^c^ (0.87–0.91)	0.87^c^ (0.84–0.89)
Yes, no	1.28^c^ (1.16–1.40)	1.23^c^ (1.12–1.35)	0.93 (0.85–1.03)	1.32^c^ (1.20–1.44)	1.29^c^ (1.17–1.42)	1.29^c^ (1.16–1.44)	1.18^c^ (1.07–1.30)	1.06 (0.96–1.17)	1.12^b^ (1.02–1.23)
eth#intpv									
No, no	1 [Reference]	1 [Reference]	1 [Reference]	1 [Reference]	1 [Reference]	1 [Reference]	1 [Reference]	1 [Reference]	1 [Reference]
No, yes	0.86^c^ (0.84–0.87)	0.77^c^ (0.76–0.79)	0.89^c^ (0.87–0.91)	0.92^c^ (0.90–0.94)	0.87^c^ (0.85–0.89)	0.94^c^ (0.91–0.96)	1.00 (0.98–1.03)	1.09^c^ (1.07–1.11)	0.95^c^ (0.93–0.97)
Yes, no	1.00 (0.92–1.09)	1.14^c^ (1.05–1.24)	1.05 (0.96–1.14)	1.15^c^ (1.06–1.25)	1.12^c^ (1.03–1.22)	1.10^a^ (1.00–1.22)	1.26^c^ (1.16–1.37)	1.20^c^ (1.10–1.31)	1.24^c^ (1.14–1.34)
eth#mari									
No, no	1 [Reference]	1 [Reference]	1 [Reference]	1 [Reference]	1 [Reference]	1 [Reference]	1 [Reference]	1 [Reference]	1 [Reference]
No, yes	0.80^c^ (0.78–0.82)	1.03^b^ (1.01–1.06)	2.54^c^ (2.48–2.61)	0.95^c^ (0.93–0.97)	0.99 (0.96–1.01)	0.88^c^ (0.86–0.91)	0.91^c^ (0.89–0.94)	1.02^a^ (1.00–1.05)	0.90^c^ (0.88–0.92)
Yes, no	1.03 (0.95–1.11)	0.91^b^ (0.84–0.98)	0.45^c^ (0.42–0.49)	1.03 (0.95–1.11)	1.01 (0.94–1.09)	1.08 (0.98–1.18)	1.05 (0.97–1.13)	1.07 (0.99–1.16)	1.09^b^ (1.01–1.18)
eth#zon1									
No, no	1 [Reference]	1 [Reference]	1 [Reference]	1 [Reference]	1 [Reference]	1 [Reference]	1 [Reference]	1 [Reference]	1 [Reference]
No, yes	0.95 (0.86–1.05)	1.59^c^ (1.45–1.74)	1.40^c^ (1.27–1.55)	0.62^c^ (0.56–0.68)	0.99 (0.90–1.08)	0.28^c^ (0.25–0.32)	0.49^c^ (0.44–0.53)	0.95 (0.87–1.04)	0.64^c^ (0.59–0.71)
Yes, no	0.60^c^ (0.52–0.69)	1.25^c^ (1.09–1.45)	0.82^b^ (0.70–0.96)	1.34^c^ (1.15–1.55)	1.23^c^ (1.07–1.42)	1.13 (0.94–1.36)	1.59^c^ (1.37–1.85)	1.61^c^ (1.39–1.86)	1.03 (0.89–1.19)
eth#zon2									
No, no	1 [Reference]	1 [Reference]	1 [Reference]	1 [Reference]	1 [Reference]	1 [Reference]	1 [Reference]	1 [Reference]	1 [Reference]
No, yes	0.70^c^ (0.63–0.77)	1.92^c^ (1.76–2.10)	0.78^c^ (0.71–0.86)	0.57^c^ (0.53–0.63)	1.01 (0.93–1.10)	0.25^c^ (0.22–0.28)	0.38^c^ (0.35–0.42)	0.70^c^ (0.64–0.76)	0.46^c^ (0.42–0.51)
Yes, no	1.08 (0.95–1.23)	1.15^b^ (1.01–1.30)	1.61^c^ (1.41–1.84)	1.91^c^ (1.67–2.19)	1.30^c^ (1.15–1.48)	1.67^c^ (1.40–2.00)	3.38^c^ (2.92–3.91)	2.40^c^ (2.11–2.73)	2.01^c^ (1.75–2.31)
eth#zon3									
No, no	1 [Reference]	1 [Reference]	1 [Reference]	1 [Reference]	1 [Reference]	1 [Reference]	1 [Reference]	1 [Reference]	1 [Reference]
No, yes	0.59^c^ (0.54–0.65)	2.06^c^ (1.89–2.24)	0.90^b^ (0.82–0.99)	0.56^c^ (0.51–0.61)	0.91^b^ (0.83–0.99)	0.19^c^ (0.17–0.21)	0.35^c^ (0.32–0.39)	0.63^c^ (0.57–0.68)	0.42^c^ (0.38–0.46)
Yes, no	1.08 (0.96–1.21)	0.82^c^ (0.73–0.93)	1.85^c^ (1.65–2.08)	1.73^c^ (1.53–1.94)	1.23^c^ (1.09–1.38)	1.98^c^ (1.69–2.33)	3.09^c^ (2.72–3.50)	3.50^c^ (3.11–3.94)	2.15^c^ (1.90–2.44)
eth#zon4									
No, no	1 [Reference]	1 [Reference]	1 [Reference]	1 [Reference]	1 [Reference]	1 [Reference]	1 [Reference]	1 [Reference]	1 [Reference]
No, yes	0.60^c^ (0.55–0.66)	1.88^c^ (1.73–2.05)	1.17^c^ (1.07–1.28)	0.72^c^ (0.66–0.78)	1.09^b^ (1.01–1.19)	0.29^c^ (0.26–0.32)	0.66^c^ (0.61–0.72)	1.13^c^ (1.04–1.23)	0.63^c^ (0.57–0.68)

*eth* ethnicity; *agg* age group; *edu* education level; *huk *hukou; *intpv* interprovincial floating; *mar* first marriage; *zon1* Pearl River Delta; *zon2* Yangtze River Delta; *zon3* Bohai Rim; *zon4* other economic zone

^a–c^10, 5, and 1%, respectively

Illiterate EMFP had less likelihood to obtain health knowledge of occupational diseases (aOR = 0.53, 95% CI 0.46–0.62), nutrition (aOR = 0.60, 95% CI 0.53–0.69), reproduction (aOR = 0.60, 95% CI 0.53–0.69), chronic diseases (aOR = 0.61, 95% CI 0.53–0.70), smoking control (aOR = 0.61, 95% CI 0.53–0.69), mental disorders (aOR = 0.72, 95% CI 0.60–0.86), tuberculosis (aOR = 0.86, 95% CI 0.76–0.98), STD/AIDS (aOR = 0.61, 95% CI 0.53–0.69), and other infectious diseases (aOR = 0.77, 95% CI 0.67–0.88) as compared to illiterate HMFP.

EMFP with non-agricultural hukou had more likelihood to obtain health knowledge of occupational diseases (aOR = 1.28, 95% CI 1.16–1.40), nutrition (aOR = 1.23, 95% CI 1.12–1.35), chronic diseases (aOR = 1.32, 95% CI 1.20–1.44), smoking control (aOR = 1.29, 95% CI 1.17–1.42), mental disorders (aOR = 1.29, 95% CI 1.16–1.44), tuberculosis (aOR = 1.18, 95% CI 1.07–1.30), and other infectious diseases (aOR = 1.12, 95% CI 1.02–1.23) as compared to HMFP with non-agricultural hukou.

EMFP not interprovincial floating had more likelihood to obtain health knowledge of nutrition (aOR = 1.14, 95% CI 1.05–1.24), chronic diseases (aOR = 1.15, 95% CI 1.06–1.25), smoking control (aOR = 1.12, 95% CI 1.03–1.22), mental disorders (aOR = 1.10, 95% CI 1.00–1.22), tuberculosis (aOR = 1.26, 95% CI 1.16–1.37), STD/AIDS (aOR = 1.20, 95% CI 1.10–1.31), and other infectious diseases (aOR = 1.24, 95% CI 1.14–1.34) compared to HMFP without interprovincial floating.

EMFP without first marriage had less likelihood to obtain health knowledge of nutrition (aOR = 0.91, 95% CI 0.84–0.98), reproduction (aOR = 0.45, 95% CI 0.42–0.49) and had more likelihood to obtain health knowledge of other infectious diseases (aOR = 1.09, 95% CI 1.01–1.18) as compared to HMFP without first marriage.

Geographically, EMFP not living in Pearl River Delta had less likelihood to obtain health knowledge of occupational diseases (aOR = 0.60, 95% CI 0.52–0.69) and reproduction (aOR = 0.82, 95% CI 0.70–0.96) and more likelihood to obtain health knowledge of nutrition (aOR = 1.25, 95% CI 1.09–1.45), chronic diseases (aOR = 1.34, 95% CI 1.15–1.55), smoking control (aOR = 1.23, 95% CI 1.07–1.42), tuberculosis (aOR = 1.59, 95% CI 1.37–1.85), and STD/AIDS (aOR = 1.61, 95% CI 1.39–1.86) as compared to HMFP not living in Pearl River Delta.

EMFP not living in Yangtze River Delta had more likelihood to obtain health knowledge of nutrition (aOR = 1.15, 95% CI 1.01–1.30), reproduction (aOR = 1.61, 95% CI 1.41–1.84), chronic diseases (aOR = 1.91, 95% CI 1.67–2.19), smoking control (aOR = 1.30, 95% CI 1.15–1.48), mental disorders (aOR = 1.67, 95% CI 1.40–2.00), tuberculosis (aOR = 3.38, 95% CI 2.92–3.91), STD/AIDS (aOR = 2.40, 95% CI 2.11–2.73), and other infectious diseases (aOR = 2.01, 95% CI 1.75–2.31) as compared to HMFP not living in Yangtze River Delta.

EMFP not living in Bohai Rim had less likelihood to obtain health knowledge of nutrition (aOR = 0.82, 95% CI 0.73–0.93) and had more likelihood to obtain health knowledge of reproduction (aOR = 1.85, 95% CI 1.65–2.08), chronic diseases (aOR = 1.73, 95% CI 1.53–1.94), smoking control (aOR = 1.23, 95% CI 1.09–1.38), mental disorders (aOR = 1.98, 95% CI 1.69–2.33), tuberculosis (aOR = 3.09, 95% CI 2.72–3.50), STD/AIDS (aOR = 3.50, 95% CI 3.11–3.94), and other infectious diseases (aOR = 2.15, 95% CI 1.90–2.44) as compared to HMFP not living in Bohai Rim. Thus, there existed disparities in association between ethnicity and health knowledge categories among EMFP. Accordingly, Hypothesis 1 was accepted.

### Association between ethnicity and health knowledge sources

In Table 4, as compared to young HMFP, EMFP was more likely to use radio/TV programmes and bulletin board and less likely to use lecture, face-to-face consultation, online education, community advocacy, and SMS/WeChat to obtain health knowledge.

**Table 4:** Logistic regression on health knowledge sources, Odds Ratio [95% Conf. Interval] (N = 189,345)

	Lecture	Books/magazine/CD	Radio/TV programmes	Face-to-face consultation	Online education	Community advocacy	Bulletin board	SMS/WeChat
eth#agg								
NO, young	1 [Reference]	1 [Reference]	1 [Reference]	1 [Reference]	1 [Reference]	1 [Reference]	1 [Reference]	1 [Reference]
NO, middle	1.02^a^ (1.00–1.04)	0.86^c^ (0.84–0.88)	1.09^c^ (1.06–1.12)	0.98^a^ (0.95–1.00)	0.60^c^ (0.59–0.62)	1.06^c^ (1.04–1.09)	1.04^c^ (1.01–1.07)	0.67^c^ (0.66–0.69)
NO, old	1.26^c^ (1.17–1.35)	0.51^c^ (0.48–0.55)	1.58^c^ (1.44–1.73)	1.05 (0.97–1.13)	0.14^c^ (0.13–0.15)	1.22^c^ (1.14–1.30)	0.76^c^ (0.70–0.82)	0.14^c^ (0.13–0.16)
Yes, young	0.08^c^ (0.06–0.10)	0.36^c^ (0.28–0.47)	2.57^c^ (1.88–3.50)	0.04^c^ (0.03–0.05)	0.65^c^ (0.50–0.84)	0.13^c^ (0.10–0.17)	1.31 (0.93–1.83)	0.80^a^ (0.62–1.03)
Yes, middle	0.08^c^ (0.06–0.10)	0.33^c^ (0.26–0.42)	3.01^c^ (2.23–4.07)	0.03^c^ (0.02–0.05)	0.44^c^ (0.34–0.56)	0.13^c^ (0.10–0.17)	1.32^a^ (0.96–1.83)	0.58^c^ (0.46–0.75)
Yes, old	0.09^c^ (0.06–0.12)	0.23^c^ (0.16–0.32)	3.81^c^ (2.52–5.77)	0.04^c^ (0.03–0.06)	0.10^c^ (0.06–0.15)	0.16^c^ (0.11–0.22)	0.74 (0.49–1.12)	0.15^c^ (0.11–0.21)
eth#gender								
No, no	1 [Reference]	1 [Reference]	1 [Reference]	1 [Reference]	1 [Reference]	1 [Reference]	1 [Reference]	1 [Reference]
No, yes	0.89^c^ (0.87–0.90)	1.04^c^ (1.02–1.06)	0.99 (0.97–1.02)	0.85^c^ (0.83–0.87)	1.10^c^ (1.08–1.12)	0.93^c^ (0.91–0.95)	0.95^c^ (0.92–0.97)	1.09^c^ (1.07–1.11)
Yes, no	1.14^c^ (1.06–1.23)	0.93^b^ (0.87–1.00)	1.08^a^ (0.99–1.17)	1.15^c^ (1.07–1.24)	0.89^c^ (0.83–0.96)	1.13^c^ (1.05–1.20)	0.99 (0.90–1.08)	0.88^c^ (0.82–0.94)
eth#edu								
No, no	1 [Reference]	1 [Reference]	1 [Reference]	1 [Reference]	1 [Reference]	1 [Reference]	1 [Reference]	1 [Reference]
No, yes	1.43^c^ (1.30–1.57)	1.82^c^ (1.65–1.99)	1.00 (0.90–1.12)	1.03 (0.94–1.14)	2.73^c^ (2.44–3.05)	1.31^c^ (1.20–1.43)	1.99^c^ (1.81–2.18)	2.35^c^ (2.14–2.57)
Yes, no	0.81^c^ (0.69–0.95)	0.45^c^ (0.39–0.53)	0.87 (0.73–1.04)	0.85^b^ (0.73–1.00)	0.42^c^ (0.35–0.51)	0.74^c^ (0.64–0.86)	0.52^c^ (0.44–0.62)	0.63^c^ (0.54–0.73)
eth#huk								
No, no	1 [Reference]	1 [Reference]	1 [Reference]	1 [Reference]	1 [Reference]	1 [Reference]	1 [Reference]	1 [Reference]
No, yes	0.75^c^ (0.73–0.77)	0.78^c^ (0.76–0.80)	0.99 (0.96–1.03)	0.97^b^ (0.94–0.99)	0.69^c^ (0.67–0.71)	0.89^c^ (0.87–0.91)	0.91^c^ (0.88–0.94)	0.86^c^ (0.83–0.88)
Yes, no	1.32^c^ (1.19–1.46)	1.34^c^ (1.22–1.48)	1.00 (0.88–1.13)	0.86^c^ (0.77–0.96)	1.50^c^ (1.36–1.66)	1.20^c^ (1.09–1.32)	1.07 (0.93–1.23)	1.46^c^ (1.32–1.61)
eth#intpv								
No, no	1 [Reference]	1 [Reference]	1 [Reference]	1 [Reference]	1 [Reference]	1 [Reference]	1 [Reference]	1 [Reference]
No, yes	0.92^c^ (0.90–0.94)	0.95^c^ (0.93–0.97)	1.04^c^ (1.02–1.07)	0.96^c^ (0.94–0.99)	0.94^c^ (0.92–0.96)	0.90^c^ (0.88–0.92)	0.95^c^ (0.93–0.98)	0.99 (0.97–1.01)
Yes, no	1.12^b^ (1.02–1.23)	1.07 (0.98–1.17)	1.03 (0.92–1.15)	1.23^c^ (1.12–1.35)	0.96^c^ (0.87–1.05)	1.19^c^ (1.09–1.30)	1.12^a^ (0.99–1.27)	0.86^c^ (0.79–0.94)
eth#mar								
No, no	1 [Reference]	1 [Reference]	1 [Reference]	1 [Reference]	1 [Reference]	1 [Reference]	1 [Reference]	1 [Reference]
No, yes	0.91^c^ (0.89–0.94)	0.95^c^ (0.93–0.98)	1.11^c^ (1.08–1.14)	1.24^c^ (1.21–1.28)	0.90^c^ (0.88–0.93)	0.98 (0.96–1.01)	1.10^c^ (1.07–1.14)	0.89^c^ (0.87–0.92)
Yes, no	1.10^b^ (1.01–1.20)	1.15^c^ (1.06–1.24)	1.02 (0.92–1.13)	0.78^c^ (0.72–0.86)	1.21^c^ (1.11–1.31)	1.00 (0.92–1.08)	1.09 (0.97–1.22)	1.19^c^ (1.09–1.29)
eth#zon1								
No, no	1 [Reference]	1 [Reference]	1 [Reference]	1 [Reference]	1 [Reference]	1 [Reference]	1 [Reference]	1 [Reference]
No, yes	0.40^c^ (0.36–0.45)	0.58 ^c^ (0.53–0.65)	3.42^c^ (3.03–3.86)	0.27^c^ (0.24–0.30)	0.61^c^ (0.54–0.69)	0.56^c^ (0.50–0.62)	2.88^c^ (2.57–3.23)	0.84^c^ (0.76–0.94)
Yes, no	1.80^c^ (1.50–2.15)	1.22^c^ (1.05–1.43)	1.20^a^ (0.99–1.44)	2.25^c^ (1.86–2.72)	0.93 (0.80–1.09)	1.44^c^ (1.23–1.68)	1.28^b^ (1.03–1.57)	1.27^c^ (1.09–1.48)
eth#zon2								
No, no	1 [Reference]	1 [Reference]	1 [Reference]	1 [Reference]	1 [Reference]	1 [Reference]	1 [Reference]	1 [Reference]
No, yes	0.37^c^ (0.33–0.41)	0.56^c^ (0.51–0.62)	4.02^c^ (3.58–4.52)	0.26^c^ (0.23–0.28)	0.60^c^ (0.54–0.68)	0.46^c^ (0.42–0.51)	1.96–2.43	0.95 (0.86–1.05)
Yes, no	2.37^c^ (1.99–2.82)	1.16^b^ (1.01–1.33)	0.84^a^ (0.70–1.01)	2.50^c^ (2.09–2.99)	1.05 (0.91–1.22)	1.80^c^ (1.55–2.09)	1.71^c^ (1.43–2.05)	1.04 (0.91–1.20)
eth#zon3								
No, no	1 [Reference]	1 [Reference]	1 [Reference]	1 [Reference]	1 [Reference]	1 [Reference]	1 [Reference]	1 [Reference]
No, yes	0.54^c^ (0.48–0.59)	0.52^c^ (0.47–0.58)	2.59^c^ (2.31–2.91)	0.31^c^ (0.28–0.34)	0.47^c^ (0.41–0.53)	0.44^c^ (0.40–0.48)	1.83^c^ (1.65–2.04)	0.74^c^ (0.67–0.82)
Yes, no	1.26^c^ (1.11–1.44)	1.60^c^ (1.42–1.82)	1.49^c^ (1.29–1.72)	2.42^c^ (2.08–2.82)	1.22^c^ (1.08–1.39)	1.92^c^ (1.69–2.18)	2.30^c^ (1.98–2.68)	1.56^c^ (1.39–1.77)
eth#zon4								
No, no	1 [Reference]	1 [Reference]	1 [Reference]	1 [Reference]	1 [Reference]	1 [Reference]	1 [Reference]	1 [Reference]
No, yes	0.48^c^ (0.43–0.53)	0.64^c^ (0.58–0.71)	3.44^c^ (3.07–3.85)	0.40^c^ (0.37–0.45)	0.56^c^ (0.50–0.63)	0.63^c^ (0.58–0.69)	3.16^c^ (2.85–3.50)	0.93 (0.84–1.03)

*eth* ethnicity; *agg* age group; *edu* education level; *huk *hukou; *intpv* interprovincial floating; *mar* first marriage; *zon1* Pearl River Delta; *zon2* Yangtze River Delta; *zon3* Bohai Rim; *zon4* other economic zone

^a–c^10, 5, and 1%, respectively

As compared to female HMFP, female EMFP was more likely to use lecture (aOR = 1.14, 95% CI 1.06–1.23), radio/TV programmes (aOR = 1.08, 95% CI 0.99–1.17), face-to-face consultation (aOR = 1.15, 95% CI 1.07–1.24), and community advocacy (aOR = 1.13, 95% CI 1.05–1.20) and were less likely to use books/magazine/CD (aOR = 0.93, 95% CI 0.87–1.00), online education (aOR = 0.89, 95% CI 0.83–0.96), and SMS/WeChat (aOR = 0.88, 95% CI 0.82–0.94) to obtain health knowledge.

As compared to Illiterate HMFP, illiterate EMFP was more likely to obtain health knowledge with lecture (aOR = 0.81, 95% CI 0.69–0.95), books/magazine/CD (aOR = 0.45, 95% CI 0.39–0.53), face-to-face consultation (aOR = 0.85, 95% CI 0.73–1.00), online education (aOR = 0.42, 95% CI 0.35–0.51), community advocacy (aOR = 0.74, 95% CI 0.64–0.86), bulletin board (aOR = 0.52, 95% CI 0.44–0.62), and SMS/WeChat (aOR = 0.63, 95% CI 0.54–0.73).

As compared to HMFP with non-agricultural hukou, EMFP with non-agricultural hukou was more likely to use lecture (aOR = 1.32, 95% CI 1.19–1.46), books/magazine/CD (aOR = 1.34, 95% CI 1.22–1.48), online education (aOR = 1.50, 95% CI 1.36–1.66), community advocacy (aOR = 1.20, 95% CI 1.09–1.32), and SMS/WeChat (aOR = 1.46, 95% CI 1.32–1.61) and were less likely to use face-to-face consultation (aOR = 0.86, 95% CI 0.77–0.96) to obtain health knowledge.

Compared with HMFP without interprovincial floating, EMFP not interprovincial floating was more likely to use lecture (aOR = 1.12, 95% CI 1.02–1.23), face-to-face consultation (aOR = 1.23, 95% CI 1.12–1.35), community advocacy (aOR = 1.19, 95% CI 1.09–1.30), bulletin board (aOR = 1.12, 95% CI 0.99–1.27), and were less likely to use online education (aOR = 0.96, 95% CI 0.87–1.05) and SMS/WeChat (aOR = 0.86, 95% CI 0.79–0.94) to obtain health knowledge.

As compared to HMFP without first marriage, EMFP without first marriage was more likely to use lecture (aOR = 1.10, 95% CI 1.01–1.20), books/magazine/CD (aOR = 1.15, 95% CI 1.06–1.24), online education (aOR = 1.21, 95% CI 1.11–1.31), and SMS/WeChat (aOR = 1.19, 95% CI 1.09–1.29) and were less likely to use face-to-face consultation (aOR = 0.78, 95% CI 0.72–0.86) to obtain health knowledge.

As compared to HMFP not living in Pearl River Delta, EMFP not living in Pearl River Delta was more likely to use lecture (aOR = 1.80, 95% CI 1.50–2.15), books/magazine/CD (aOR = 1.22, 95% CI 1.05–1.43), radio/TV programmes (aOR = 1.20, 95% CI 0.99–1.44), face-to-face consultation (aOR = 2.25, 95% CI 1.86–2.72), community advocacy (aOR = 1.44, 95% CI 1.23–1.68), bulletin board (aOR = 1.28, 95% CI 1.03–1.57), and SMS/WeChat (aOR = 1.27, 95% CI 1.09–1.48) to obtain health knowledge.

As compared to HMFP not living in Yangtze River Delta, EMFP not living in Yangtze River Delta was more likely to use lecture (aOR = 2.37, 95% CI 1.99–2.82), books/magazine/CD (aOR = 1.16, 95% CI 1.01–1.33), face-to-face consultation (aOR = 2.50, 95% CI 2.09–2.99), community advocacy (aOR = 1.80, 95% CI 1.55–2.09), and bulletin board (aOR = 1.71, 95% CI 1.43–2.05) and were less likely to use radio/TV programmes (aOR = 0.84, 95% CI 0.70–1.01) to obtain health knowledge.

As compared to HMFP not living in Bohai Rim, EMFP not living in Bohai Rim was more likely to use lecture (aOR = 1.26, 95% CI 1.11–1.44), books/magazine/CD (aOR = 1.60, 95% CI 1.42–1.82), radio/TV programmes (aOR = 1.49, 95% CI 1.29–1.72), face-to-face consultation (aOR = 2.42, 95% CI 2.08–2.82), online education (aOR = 1.22, 95% CI 1.08–1.39), community advocacy (aOR = 1.92, 95% CI 1.69–2.18), bulletin board (aOR = 2.30, 95% CI 1.98–2.68), and SMS/WeChat (aOR = 1.56, 95% CI 1.39–1.77) to obtain health knowledge. Thus, Hypothesis 2 was accepted.

### Correlations between health information categories and sources

In Table 5, most of correlations were weak (positive, > 0.20 and < 0.40). Likewise, rhos between a specific relationship in EMFP and HMFP in a specific zone were not similar. But, there was zero correlation between knowledge of nutrition and face-to-face consultation in EMFP in Yangtze River Delta. There were negative correlation between knowledge of smoking control and lecture and correlation between knowledge of smoking control and face-to-face consultation in EMFP in Yangtze River Delta. Furthermore in EMFP, rho between knowledge of mental disorders and online education in other economic zone, rho between knowledge of tuberculosis and radio/TV programmes in Pearl River Delta, rho between knowledge of STD/AIDS and online education in Bohai Rim, rho between knowledge of other infectious diseases and community advocacy in other economic zone were ≥ 0.50. Simultaneously, the other moderate correlations in EMFP were reported. For example, correlations between knowledge of occupational diseases and lecture in Pearl River Delta, Yangtze River Delta, and other economic zone were moderate (rhos > 0.40). Thus, EMFP mainly used lecture to access occupational diseases information in the three economic zones. Thus, Hypothesis 3 was rejected.

**Table 5:** Rho values of combinations of health knowledge categories and sources in four economic zones

Category	Source	Pearl River Delta				Yangtze River Delta				Bohai Rim				Other economic zone			
		EMFP		HMFP		EMFP		HMFP		EMFP		HMFP		EMFP		HMFP	
		Rho	SE	Rho	SE	Rho	SE	Rho	SE	Rho	SE	Rho	SE	Rho	SE	Rho	SE
Occupational diseases	Lecture	**0.44**	**0.05**	0.42	0.01	**0.41**	**0.05**	0.36	0.01	0.35	0.04	0.42	0.01	**0.47**	**0.01**	0.45	0.00
	Books/magazine/CD	0.35	0.05	0.33	0.01	0.30	0.04	0.32	0.01	0.33	0.04	0.38	0.01	0.39	0.01	0.36	0.00
	Radio/TV	0.34	0.05	0.19	0.02	0.11	0.05	0.14	0.01	0.19	0.05	0.16	0.01	0.22	0.02	0.23	0.01
	Face-to-face consultation	0.17	0.06	0.25	0.01	0.26	0.05	0.25	0.01	0.23	0.05	0.32	0.01	0.37	0.01	0.31	0.00
	Online education	0.35	0.05	0.30	0.01	0.38	0.04	0.31	0.01	**0.40**	**0.04**	0.36	0.01	**0.48**	**0.01**	0.38	0.00
	Community advocacy	0.26	0.05	0.25	0.01	0.37	0.04	0.32	0.01	0.20	0.04	0.30	0.01	0.33	0.01	0.34	0.00
	Bulletin board	0.12	0.06	0.26	0.02	0.32	0.05	0.26	0.01	0.22	0.05	0.24	0.01	0.29	0.02	0.28	0.01
	SMS/WeChat	0.33	0.05	0.27	0.01	0.34	0.04	0.27	0.01	0.30	0.04	0.30	0.01	0.35	0.01	0.30	0.00
Nutrition	Lecture	0.29	0.06	0.28	0.01	0.23	0.06	0.21	0.01	0.18	0.05	0.28	0.01	0.39	0.01	0.32	0.01
	Books/magazine/CD	**0.40**	**0.05**	0.43	0.01	**0.40**	**0.04**	0.37	0.01	0.32	0.04	0.37	0.01	**0.46**	**0.01**	0.42	0.00
	Radio/TV	0.37	0.05	0.33	0.01	0.39	0.05	0.36	0.01	0.24	0.05	0.34	0.01	0.35	0.02	0.35	0.01
	Face-to-face consultation	0.13	0.06	0.24	0.02	**0.00**	**0.06**	0.17	0.01	0.16	0.05	0.21	0.01	0.31	0.01	0.23	0.01
	Online education	0.35	0.05	0.41	0.01	0.39	0.04	0.35	0.01	0.19	0.05	0.35	0.01	**0.47**	**0.01**	0.39	0.00
	Community advocacy	0.27	0.05	0.30	0.01	0.32	0.05	0.32	0.01	0.31	0.05	0.27	0.01	0.39	0.01	0.33	0.00
	Bulletin board	0.22	0.06	0.19	0.02	0.18	0.05	0.22	0.01	0.14	0.05	0.18	0.01	0.30	0.02	0.24	0.01
	SMS/WeChat	0.39	0.05	0.39	0.01	**0.46**	**0.04**	0.35	0.01	0.34	0.04	0.29	0.01	0.35	0.01	0.35	0.00
Reproduction	Lecture	0.11	0.07	0.13	0.02	0.08	0.06	0.16	0.01	0.32	0.04	0.36	0.01	0.23	0.02	0.17	0.01
	Books/magazine/CD	0.28	0.06	0.28	0.01	0.15	0.05	0.23	0.01	0.34	0.04	0.30	0.01	0.25	0.02	0.21	0.01
	Radio/TV	0.34	0.06	0.19	0.02	0.15	0.06	0.16	0.01	0.14	0.05	0.11	0.01	0.21	0.02	0.12	0.01
	Face-to-face consultation	0.30	0.07	0.28	0.02	0.25	0.06	0.30	0.01	**0.40**	**0.05**	0.45	0.01	0.38	0.02	0.33	0.01
	Online education	0.12	0.06	0.18	0.02	0.18	0.05	0.22	0.01	0.24	0.04	0.18	0.01	0.26	0.02	0.19	0.01
	Community advocacy	0.33	0.06	0.36	0.01	0.12	0.05	0.28	0.01	0.36	0.04	0.40	0.01	0.28	0.02	0.27	0.01
	Bulletin board	**0.47**	**0.05**	0.40	0.02	0.19	0.05	0.27	0.01	**0.45**	**0.04**	0.47	0.01	0.25	0.02	0.30	0.01
	SMS/WeChat	0.07	0.06	0.16	0.02	0.08	0.05	0.19	0.01	0.27	0.04	0.12	0.01	0.16	0.02	0.16	0.01
Chronic diseases	Lecture	0.32	0.05	0.34	0.01	0.35	0.05	0.33	0.01	0.39	0.04	0.43	0.01	**0.45**	**0.01**	0.42	0.00
	Books/magazine/CD	**0.45**	**0.04**	0.40	0.01	0.32	0.04	0.36	0.01	0.38	0.04	0.44	0.01	**0.43**	**0.01**	0.38	0.00
	Radio/TV	0.34	0.05	0.27	0.02	0.14	0.06	0.22	0.01	0.28	0.05	0.28	0.01	0.29	0.02	0.29	0.01
	Face-to-face consultation	0.26	0.06	0.36	0.01	0.34	0.05	0.35	0.01	**0.41**	**0.04**	0.41	0.01	**0.42**	**0.01**	0.38	0.00
	Online education	**0.46**	**0.04**	0.38	0.01	0.30	0.04	0.32	0.01	0.39	0.04	0.40	0.01	**0.43**	**0.01**	0.37	0.00
	Community advocacy	**0.44**	**0.05**	0.40	0.01	0.34	0.04	0.36	0.01	0.34	0.04	0.40	0.01	**0.45**	**0.01**	0.42	0.00
	Bulletin board	0.23	0.06	0.32	0.02	0.33	0.05	0.32	0.01	0.37	0.04	0.37	0.01	0.37	0.02	0.36	0.01
	SMS/WeChat	**0.47**	**0.04**	0.32	0.01	0.28	0.04	0.28	0.01	0.31	0.04	0.29	0.01	0.36	0.01	0.31	0.00
Smoking control	Lecture	0.10	0.06	0.12	0.02	**− 0.05**	**0.06**	0.05	0.01	0.13	0.05	0.16	0.01	0.24	0.02	0.16	0.01
	Books/magazine/CD	0.34	0.05	0.40	0.01	0.29	0.04	0.33	0.01	0.36	0.04	0.36	0.01	**0.41**	**0.01**	0.40	0.00
	Radio/TV	0.38	0.05	0.39	0.01	0.31	0.05	0.34	0.01	0.37	0.04	0.38	0.01	0.34	0.02	0.40	0.01
	Face-to-face consultation	0.21	0.06	0.20	0.02	**− 0.02**	**0.06**	0.08	0.01	0.12	0.05	0.17	0.01	0.18	0.02	0.15	0.01
	Online education	**0.44**	**0.05**	0.41	0.01	0.33	0.04	0.33	0.01	**0.45**	**0.04**	0.41	0.01	**0.43**	**0.01**	0.40	0.00
	Community advocacy	0.29	0.05	0.24	0.01	0.12	0.05	0.21	0.01	0.23	0.04	0.23	0.01	0.29	0.01	0.25	0.00
	Bulletin board	0.14	0.06	0.18	0.02	0.23	0.05	0.19	0.01	0.22	0.05	0.21	0.01	0.27	0.02	0.22	0.01
	SMS/WeChat	**0.44**	**0.04**	0.37	0.01	0.30	0.04	0.35	0.01	**0.40**	**0.04**	0.36	0.01	**0.41**	**0.01**	0.39	0.00
Mental disorders	Lecture	0.31	0.06	0.35	0.01	0.27	0.06	0.29	0.01	**0.41**	**0.05**	0.40	0.01	0.39	0.01	0.37	0.01
	Books/magazine/CD	**0.41**	**0.05**	0.44	0.01	**0.40**	**0.05**	0.41	0.01	**0.44**	**0.05**	0.48	0.01	**0.48**	**0.01**	0.44	0.00
	Radio/TV	**0.45**	**0.06**	0.33	0.02	0.28	0.07	0.29	0.01	0.33	0.06	0.35	0.01	0.35	0.02	0.34	0.01
	Face-to-face consultation	0.21	0.06	0.40	0.01	0.25	0.06	0.34	0.01	**0.40**	**0.05**	0.47	0.01	**0.42**	**0.01**	0.38	0.01
	Online education	**0.40**	**0.05**	0.42	0.01	0.38	0.05	0.40	0.01	**0.45**	**0.05**	0.48	0.01	**0.55**	**0.01**	0.46	0.00
	Community advocacy	**0.43**	**0.05**	0.42	0.01	**0.47**	**0.05**	0.42	0.01	**0.49**	**0.04**	0.49	0.01	**0.47**	**0.01**	0.46	0.00
	Bulletin board	0.20	0.07	0.24	0.02	0.15	0.06	0.25	0.01	0.28	0.06	0.30	0.01	0.28	0.02	0.27	0.01
	SMS/WeChat	**0.43**	**0.05**	0.40	0.01	0.36	0.05	0.35	0.01	0.39	0.05	0.38	0.01	**0.43**	**0.01**	0.38	0.01
Tuberculosis	Lecture	0.36	0.05	0.35	0.01	0.31	0.05	0.32	0.01	**0.47**	**0.04**	0.45	0.01	0.37	0.01	0.36	0.00
	Books/magazine/CD	**0.45**	**0.04**	0.40	0.01	**0.40**	**0.04**	0.38	0.01	**0.41**	**0.04**	0.44	0.01	0.38	0.01	0.38	0.00
	Radio/TV	**0.50**	**0.05**	0.27	0.02	0.12	0.06	0.22	0.01	0.28	0.05	0.26	0.01	0.30	0.02	0.30	0.01
	Face-to-face consultation	0.25	0.06	0.35	0.01	0.34	0.05	0.35	0.01	**0.46**	**0.04**	0.47	0.01	0.30	0.01	0.35	0.00
	Online education	0.36	0.05	0.33	0.01	0.38	0.04	0.35	0.01	0.35	0.04	0.41	0.01	0.35	0.01	0.36	0.00
	Community advocacy	0.38	0.05	0.40	0.01	0.38	0.05	0.40	0.01	0.37	0.04	0.45	0.01	**0.40**	**0.01**	0.42	0.00
	Bulletin board	0.27	0.06	0.32	0.02	0.33	0.05	0.33	0.01	0.39	0.05	0.40	0.01	0.38	0.02	0.40	0.01
	SMS/WeChat	**0.43**	0.05	0.33	0.01	0.36	0.05	0.33	0.01	0.26	0.04	0.32	0.01	0.33	0.01	0.32	0.00
STD/AIDS	Lecture	0.18	0.06	0.18	0.01	0.09	0.05	0.14	0.01	0.37	0.04	0.37	0.01	0.31	0.02	0.22	0.01
	Books/magazine/CD	0.39	0.05	0.38	0.01	0.39	0.04	0.40	0.01	**0.42**	**0.04**	0.43	0.01	0.37	0.01	0.40	0.00
	Radio/TV	0.39	0.05	0.32	0.01	0.34	0.05	0.32	0.01	0.36	0.04	0.31	0.01	0.30	0.02	0.37	0.01
	Face-to-face consultation	0.14	0.06	0.27	0.02	0.13	0.05	0.22	0.01	0.39	0.05	0.39	0.01	0.27	0.02	0.27	0.01
	Online education	**0.41**	**0.05**	0.36	0.01	0.34	0.04	0.35	0.01	**0.53**	**0.04**	0.39	0.01	0.36	0.02	0.37	0.00
	Community advocacy	**0.43**	**0.05**	0.43	0.01	0.25	0.05	0.34	0.01	0.37	0.04	0.41	0.01	0.36	0.01	0.40	0.00
	Bulletin board	**0.40**	**0.05**	0.32	0.02	0.33	0.05	0.31	0.01	**0.45**	**0.04**	0.43	0.01	**0.40**	**0.02**	0.36	0.01
	SMS/WeChat	**0.40**	**0.05**	0.31	0.01	0.33	0.04	0.37	0.01	**0.44**	**0.04**	0.34	0.01	0.31	0.02	0.36	0.00
Other infectious diseases	Lecture	0.17	0.06	0.23	0.01	0.16	0.05	0.25	0.01	0.25	0.04	0.33	0.01	0.37	0.01	0.30	0.00
	Books/magazine/CD	**0.40**	**0.05**	0.39	0.01	0.35	0.04	0.34	0.01	0.24	0.04	0.39	0.01	0.37	0.01	0.37	0.00
	Radio/TV	0.38	0.05	0.32	0.01	0.33	0.05	0.31	0.01	0.27	0.05	0.31	0.01	0.31	0.02	0.34	0.01
	Face-to-face consultation	0.20	0.06	0.30	0.01	0.24	0.05	0.25	0.01	0.30	0.05	0.33	0.01	0.36	0.01	0.31	0.00
	Online education	0.37	0.05	0.33	0.01	0.28	0.05	0.30	0.01	0.33	0.04	0.38	0.01	0.37	0.01	0.35	0.00
	Community advocacy	**0.48**	**0.04**	0.42	0.01	**0.46**	**0.04**	0.42	0.01	**0.44**	**0.04**	0.48	0.01	**0.54**	**0.01**	0.49	0.00
	Bulletin board	0.27	0.06	0.25	0.02	0.22	0.05	0.27	0.01	0.35	0.05	0.30	0.01	0.32	0.02	0.32	0.01
	SMS/WeChat	0.37	0.05	0.33	0.01	0.28	0.05	0.31	0.01	0.29	0.04	0.34	0.01	0.34	0.01	0.33	0.00
N		940		13,013		1212		28,345		1355		31,350		11,089		102,041	

Bold values denote rhos (> or = 0.40) and their standard errors (SE)

## Discussion

This study identified the associations of ethnicity with health knowledge categories and sources which varied by geodemographic factors. In particular, EMFP heavily relied on online health information to acquire health knowledge. Pearl River Delta, Yangtze River Delta, and Bohai Rim had significant associations with parts of health knowledge categories and sources among EMFP. This indicated that there was regional inequity of health knowledge transmission for EMFP in China. Lecture, books/magazine/CD, face-to-face consultation, online education, community advocacy, and bulletin boards must be the primary methods of delivering health education among the floating population in China. The research results can also provide a better reference for the governmental health information provision from the perspective of EMFP.

Regarding level of health knowledge, the findings in this study were in accord with prior studies. For example, a study in Shanghai found that most the floating population had an inadequate knowledge of tuberculosis and their education level was associated with the ways of obtaining knowledge [62]. Even worse, another study reported low level of health information literacy of Chinese residents [63].

With respect to ethnic disparities in level of health knowledge, the findings in this study were in line with prior studies. For example, a study indicated many Yi ethnicity women were lack of knowledge of antenatal care and HIV prevention [64]. Compared to the Han Chinese, another study indicated that the other ethnic groups had lower cervical cancer knowledge levels [65]. Thus, observed ethnic differences in health knowledge may be related to governmental factors, environment, cultural customs, or to potential combinations of these factors.

Considering health information categories, the findings in this study were in agreement with prior studies. For instance, a study showed significant disparity of the rates of Tuberculosis education among the seven regions in China [66]. A meta-analysis reported poor HIV-related sexual knowledge among the floating population [67]. There were obvious differences regarding health information sources related to cardiovascular diseases between Hui Muslims and Han people [68].

Considering geodemographic factors, the findings in this study were in congruence with prior studies. For instance, sociodemographic factors were associated with the types of online health information sought among the general Chinese population [69]. Preferred sources of health information also varied by age and educational level [70]. Additionally, a cross-sectional study identified there was a social gradient for health information literacy among urban older adults aged 60 + years in Western China [71]. Similarly, a cross-sectional survey indicated parental education and socioeconomic status were significantly associated with obtaining health information among undergraduate nursing students in a medical university in Chongqing, China [72].

Poor health awareness in this study could be explained indirectly by inferior strength of geodemographic factors in place of departure and accuracy of health media. For example, a descriptive study in a remote region of China showed that utilization of maternal health care services was associated with a range of social, economic, cultural and geographic factors [73]. A systematic review reported that traditional beliefs, low levels of education, reimbursement difficulties, and language barriers limited the willingness of ethnic minority women to use maternal health services [74]. Another cross-sectional study concluded ethnic disparities in benefits distribution of government healthcare subsidies in rural Chinese ethnic minority areas [75]. Simultaneously, the quality of online health information about breast cancer from Chinese language websites was poor [76]. Because of illiteracy among the Chinese professionalism, health-related advances in newspapers were lack of accuracy [77]. An empirical evidence show social media use for health information might lead to a negative impact on pregnant women's mental health [78]. The primary reason was possibly that the total funding and funding per student of health professional education in China remained relatively low compared to other countries from 1998 to 2017 [79].

Some health knowledge sources was highlighted in the sample could be possibly explained by several literature. The current state of public health information acquisition via WeChat proved worrisome in China [80]. Actually, a comparative study in 26 European countries indicated there were positive or negative relationships between mobile media and the credibility of health sources [81]. EMFP used Radio/TV to obtain knowledge of mental disorders and tuberculosis in Pearl River Delta rather than Yangtze River Delta, Bohai Rim, and other economic zone. The content concerning cardiovascular diseases in Chinese television health programs could be used to communicate health information in China [82]. This possibly because Radio/TV could be still afforded for EMFP to receive health information. Meanwhile, investigations indicated that face-to-face consultation and community advocacy could be considered as a more effective intervention to promote health information quality [83, 84]. Zero and negative correlations between knowledge categories and sources might be caused by poor health education organizations and health information transmission.

### Strengths and limitations

There were two main strengths in this study. First, this study considered EMFP in the associations of geodemographic factors with health knowledge categories and sources. The effort was to report ethnic disparities in access to health knowledge of the floating population. Second, regressions with interactions could reflect the individual characteristics of EMFP in the ethnic disparities in access to health knowledge. Finally, the sample size could be representative of structure of population in China. According to Tabulation on 2010 Population Census of the People’s Republic of China (http://www.stats.gov.cn/tjsj/pcsj/rkpc/6rp/indexch.htm), Han majority accounted for 91.60% in the total population, ethnic minority accounted for 8.40% in the total population. Thus, the sample size of ethnic minority by and large accorded with proportion of total population in China.

There were three main limitations in this study. First, whether the sample was from non-minority regions or ethnic minority regions was not defined in the questionnaire. Thus, comparative analyses between non-minority regions and ethnic minority regions regarding policy interventions could not be conducted. Compared with non-minority regions, worse spatial healthcare access, inequality in access to doctors and health professionals, and uneven balance among primary, secondary, and tertiary hospitals were documented in a study in ethnic minority region in Sichuan, China [85]. Second, medical and clinical measurements were defined in the questionnaire. Thus, biomedical explanations for the main associations could not be obtained. For example, a cross-sectional observational study identified ethnic differences in body composition and obesity-related risk factors between Chinese and white males living in China [86]. Finally, some associations of interest were not reported by Poisson and logistic regressions because of collinearity.

### Policy implications

Health education campaigns targeting EMFP should be actively promoted. To improve the health information literacy, high-quality health information services should be delivered to EMFP. Even Chinese college students had insufficient knowledge/skills to identify health misinformation and disinformation [87]. Thus, EMFP should not accept unregulated, inaccurate, and unactionable health information. Given the floating attributes for the population, it is important to enrich health knowledge categories and improve sources of health knowledge for the EMFP in China.

## Conclusions

In conclusions, this study showed ethnic disparities in access to health knowledge categories and sources among the sample. Geographically, this study reported weak correlations between health knowledge categories and sources in EMFP in China. Specially, this study reflected ethnic disparities with respect to inaccess to health knowledge within specific regions of China. Future interventions to control ethnic disparities and population-biased issues should address geodemographic factors.

## Data Availability

http://www.chinaldrk.org.cn/.

## Abbreviations

EMFP: Ethnic minority floating population
HMFP: Han majority floating population
STD/AIDS: Sexually transmitted diseases/acquired immunodeficiency syndrome
CI: Confidence interval
IRR: Incidence rate ratios
aOR: Adjusted odds ratio

## References

[1] Ma R (2006). Ethnic relations in contemporary China: cultural tradition and ethnic policies since 1949. Policy Soc.

[2] Feng L, Li P, Wang X, Hu Z, Ma Y, Tang W, Ben Y, Mahapatra T, Cao X, Mahapatra S, Ling M, Gou A, Wang Y, Xiao J, Hou M, Wang X, Lin B, Wang F (2014). Distribution and determinants of non communicable diseases among elderly Uyghur ethnic group in Xinjiang, China. PLoS ONE.

[3] Gu H, Li W, Yang J, Wang Y, Bo J, Liu L (2015). Hypertension prevalence, awareness, treatment and control among Han and four ethnic minorities (Uygur, Hui, Mongolian and Dai) in China. J Hum Hypertens.

[4] Guo S, Pang H, Guo H, Zhang M, He J, Yan Y, Niu Q, Muratbek Rui D, Li S, Ma R, Zhang J, Liu J, Ding Y (2015). Ethnic differences in the prevalence of high homocysteine levels among low-income rural Kazakh and Uyghur adults in far western China and its implications for preventive public health. Int J Environ Res Public Health.

[5] Gao Y, Xie X, Wang SX, Li H, Tang HZ, Zhang J, Yao H (2017). Effects of sedentary occupations on type 2 diabetes and hypertension in different ethnic groups in North West China. Diab Vasc Dis Res.

[6] Luo M, Zhao Z, He L, Su B, Liu W, Zhang G (2019). Ethnic disparity in pneumonia-specific mortality among children under 5 years of age in Sichuan Province of Western China from 2010 to 2017. BMC Public Health.

[7] Cao J, Chen J, Zhang Q, Wu J, Wang W, Zhang X, Zhao D, Zhang Q, Yang W, Chen Z (2020). Ethnic disparities in demographic, clinicopathologic and biological behaviours and prognosis of gastric cancer in northwest China. Cancer Med.

[8] Heizhati M, Wang L, Yao X, Li M, Hong J, Luo Q, Zhang D, Abulikemu S, Wu T, Li N (2020). Prevalence, awareness, treatment and control of hypertension in various ethnic groups (Hui, Kazakh, Kyrgyz, Mongolian, Tajik) in Xinjiang, Northwest China. Blood Press.

[9] Liang Z, Li Z, Ma Z (2014). Changing patterns of the floating population in China during 2000–2010. Popul Dev Rev.

[10] Cai F (1996). Causes, trends, and policy of population migration and the floating population. Chin J Popul Sci.

[11] Lu XP, Wu M (2010). Determinants of household healthcare expenditure of rural floating population in Beijing: a Tobit model approach. Beijing Da Xue Xue Bao Yi Xue Ban.

[12] Guan YQ, Zhang M, Zhang X, Zhao ZP, Huang ZJ, Li C, Wang LM (2019). Medical treatment seeking behaviors and its influencing factors in employed floating population in China. Zhonghua Liu Xing Bing Xue Za Zhi.

[13] Zhang L, Wu M (2015). Analysis on status and determinants of self-treatment of rural floating population in Beijing. Beijing Da Xue Xue Bao Yi Xue Ban.

[14] Zhou Y, Wang T, Fu J, Chen M, Meng Y, Luo Y (2019). Access to reproductive health services among the female floating population of childbearing age: a cross-sectional study in Changsha, China. BMC Health Serv Res.

[15] Su L, Sun L, Xu L (2018). Review on the prevalence, risk factors and disease Management of Hypertension among floating population in China during 1990–2016. Glob Health Res Policy.

[16] Anderson AF, Qingsi Z, Hua X, Jianfeng B (2003). China’s floating population and the potential for HIV transmission: a social-behavioural perspective. AIDS Care.

[17] Cheng-rong D, Song-jian C (2011). Study on floating minorities of China. Popul J.

[18] Jihe LI, Dongmei MA, Lan C (2013). Basic characteristics of floating urban minorities in contemporary China: a case study of Muslim groups in Central and East China. J Yunnan Natl Univ.

[19] Wu J, Wang MYL (2012). Ethnic migrant workers and emerging ethnic division in China’s urban labor market. Asian Pac Migr J.

[20] Mobius M, Rosenblat T, Wang Q (2016). Ethnic discrimination: evidence from China. Eur Econ Rev.

[21] Ouyang Y, Pinstrup-Andersen P (2012). Health inequality between ethnic minority and han populations in China. World Dev.

[22] Xu F, Binns C, Nazi G, Shi L, Zhao Y, Lee A (2006). A comparison of breastfeeding among Han, Uygur and other ethnic groups in Xinjiang, PR China. BMC Public Health.

[23] Wang XM, Wu C, Golden AR, Le C (2019). Ethnic disparities in prevalence and patterns of smoking and nicotine dependence in rural southwest China: a cross-sectional study. BMJ Open.

[24] Wang YJ, Chen XP, Chen WJ, Zhang ZL, Zhou YP, Jia Z (2020). Ethnicity and health inequalities: an empirical study based on the 2010 China survey of social change (CSSC) in Western China. BMC Public Health.

[25] Zhong B, Chen J (2021). Health information helps mitigate adolescent depression: a multivariate analysis of the links between health information use and depression management. Child Care Health Dev.

[26] Chan EYY, Lam HCY, Lo ESK, Tsang SNS, Yung TKC, Wong CKP (2019). Food-related health emergency-disaster risk reduction in rural ethnic minority communities: a pilot study of knowledge, awareness and practice of food labelling and salt-intake reduction in a Kunge community in China. Int J Environ Res Public Health.

[27] Farsi NJ, Merdad Y, Mirdad M (2020). Oral health knowledge, attitudes, and behaviors among university students in Jeddah, Saudi Arabia. Clin Cosmet Investig Dent.

[28] Kwon SJ, Im YM (2021). Sexual health knowledge and needs among young adults with congenital heart disease. PLoS ONE.

[29] Burke A, Nahin RL, Stussman BJ (2015). Limited health knowledge as a reason for non-use of four common complementary health practices. PLoS ONE.

[30] Alhaija ESA, Al-Saif EM, Taani DQ (2018). Periodontal health knowledge and awareness among subjects with fixed orthodontic appliance. Dental Press J Orthod.

[31] He Z, Cheng Z, Shao T (2016). Factors influencing health knowledge and behaviors among the elderly in rural China. Int J Environ Res Public Health.

[32] Oechsle A, Wensing M, Ullrich C, Bombana M (2020). Health knowledge of lifestyle-related risks during pregnancy: a cross-sectional study of pregnant women in Germany. Int J Environ Res Public Health.

[33] Llena C, Nakdali T, Sanz JL, Forner L (2019). Oral health knowledge and related factors among pregnant women attending to a primary care center in Spain. Int J Environ Res Public Health.

[34] Andrew L, Wallace R, Wickens N, Patel J (2021). Early childhood caries, primary caregiver oral health knowledge and behaviours and associated sociological factors in Australia: a systematic scoping review. BMC Oral Health.

[35] Bornstein S, Markos JR, Murad MH, Mauck K, Molella R (2021). Improving collaboration between public health and medicine: a timely survey of clinician public health knowledge, training, and engagement. Mayo Clin Proc Innov Qual Outcomes.

[36] Gautam A, Bhatta DN, Aryal UR (2015). Diabetes related health knowledge, attitude and practice among diabetic patients in Nepal. BMC Endocr Disord.

[37] Aburahima N, Hussein I, Kowash M, Alsalami A, Al Halabi M (2020). Assessment of paediatricians’ oral health knowledge, behaviour, and attitude in the United Arab Emirates. Int J Dent.

[38] Heslop IM, Speare R, Bellingan M, Glass BD (2020). Assessing the travel health knowledge of Australian pharmacists. Pharmacy.

[39] Yimenu DK, Adelo ES, Siraj EA (2020). Health professionals oral health knowledge and practice: unleashing the hidden challenges. J Multidiscip Healthc.

[40] Spagnolo J, Champagne F, Leduc N (2018). Mental health knowledge, attitudes, and self-efficacy among primary care physicians working in the Greater Tunis area of Tunisia. Int J Ment Health Syst.

[41] Cianetti S, Anderini P, Pagano S (2020). Oral Health knowledge level of nursing staff working in semi-intensive heart failure units. J Multidiscip Healthc.

[42] Alrashdi M, Limaki ME, Alrashidi A (2021). Oral health knowledge gaps and their impact on the role of pediatricians: a multicentric study. Int J Environ Res Public Health.

[43] Akuiyibo S, Anyanti J, Idogho O (2021). Impact of peer education on sexual health knowledge among adolescents and young persons in two North Western states of Nigeria. Reprod Health.

[44] He Y, Guo L, Liu Y (2021). Can goal-based health management improve the health knowledge, health belief and health behavior in people at high risk of stroke? A non-randomized controlled trial. Neuropsychiatr Dis Treat.

[45] Bian D, Shi Y, Tang W (2021). The influencing factors of nutrition and diet health knowledge dissemination using the WeChat official account in health promotion. Front Public Health.

[46] Qiu Y, Qin H, Ying M, Xu K, Ren J (2020). WeChat-based health education to improve health knowledge in three major infectious diseases among residents: a multicentre case-controlled protocol. BMJ Open.

[47] Mayerová D, Abbas K (2021). Childhood immunisation timeliness and vaccine confidence by health information source, maternal, socioeconomic, and geographic characteristics in Albania. BMC Public Health.

[48] Ghalavand H, Panahi S, Sedghi S (2020). Opportunities and challenges of social media for health knowledge management: a narrative review. J Educ Health Promot.

[49] Sugiyama S, Okuda M, Kinoshita T (2011). Association between visual message and health knowledge in a 4-month follow-up study at worksites. J Occup Health.

[50] Smith H, Bukirwa H, Mukasa O (2007). Access to electronic health knowledge in five countries in Africa: a descriptive study. BMC Health Serv Res.

[51] Lee YC, Wu WL (2014). The effects of situated learning and health knowledge involvement on health communications. Reprod Health.

[52] Xu S, Markson C, Costello KL, Xing CY, Demissie K, Llanos AA (2016). Leveraging social media to promote public health knowledge: example of cancer awareness via Twitter. JMIR Public Health Surveill.

[53] Mesheriakova VV, Tebb KP (2017). Effect of an iPad-based intervention to improve sexual health knowledge and intentions for contraceptive use among adolescent females at school-based health centers. Clin Pediatr.

[54] Song S, Xue X, Zhao YC, Li J, Zhu Q, Zhao M (2021). Short-video apps as a health information source for chronic obstructive pulmonary disease: information quality assessment of TikTok videos. J Med Internet Res.

[55] Kong W, Song S, Zhao YC, Zhu Q, Sha L (2021). TikTok as a health information source: assessment of the quality of information in diabetes-related videos. J Med Internet Res.

[56] Liu X, Lu J, Wang H (2017). When health information meets social media: exploring virality on Sina Weibo. Health Commun.

[57] Smolich L, Charen K, Sherman SL (2020). Health knowledge of women with a fragile X premutation: improving understanding with targeted educational material. J Genet Couns.

[58] Zhang T, Miao Y, Li L, Bian Y (2019). Awareness of HIV/AIDS and its routes of transmission as well as access to health knowledge among rural residents in Western China: a cross-sectional study. BMC Public Health.

[59] Verlinden DA, Schuller AA, Verrips GHW, Reijneveld SA (2020). Effectiveness of a short web-based film targeting parental oral health knowledge in a well-child care setting. Eur J Oral Sci.

[60] Sajaia Z (2008). “BIOPROBIT: stata module for bivariate ordered probit regression,” statistical software components S456920.

[61] Evans JD (1996). Straightforward statistics for the behavioral sciences.

[62] Zhao DH, Li HD, Xu B (2005). Knowledge of tuberculosis amongst service industry workers of the floating population in Changning district, Shanghai. Zhonghua Jie He He Hu Xi Za Zhi.

[63] Nie X, Li Y, Li L, Huang X (2014). A study on health information literacy among urban and suburban residents in six provinces in China. Zhonghua Yu Fang Yi Xue Za Zhi.

[64] Liu B, Shi D, Wang W, Nan L, Yin B, Wei C, Emu A, Zhou H, Wazha W, Zhu J, Wang S, Ma W (2019). What factors hinder ethnic minority women in rural China from getting antenatal care? A retrospective data analysis. BMJ Open.

[65] Wu E, Tiggelaar SM, Jiang T, Zhao H, Wu R, Wu R, Xu F (2018). Cervical cancer prevention-related knowledge and attitudes among female undergraduate students from different ethnic groups within China, a survey-based study. Women Health.

[66] Zhu Z, Guo M, Dong T, Gong B, Zhao X, Hu Y (2021). Do migrants receive tuberculosis education in China? Evidence from the China migrants dynamic survey. Health Educ Behav.

[67] Chen LX, Liang H, Yang XB (2012). Meta-analysis on the effects of health education towards HIV/AIDS high-risk behavior, knowledge, and related attitude among floating population in China. Zhonghua Liu Xing Bing Xue Za Zhi.

[68] Yang L, Mao Y, Jansz J (2018). Chinese urban Hui Muslims’ access to and evaluation of cardiovascular diseases-related health information from different sources. Int J Environ Res Public Health.

[69] Xiong Z, Zhang L, Li Z, Xu W, Zhang Y, Ye T (2021). Frequency of online health information seeking and types of information sought among the general Chinese population: cross-sectional study. J Med Internet Res.

[70] Hillyer GC, Schmitt KM, Lizardo M (2017). Electronic communication channel use and health information source preferences among Latinos in Northern Manhattan. J Community Health.

[71] Li C, Guo Y (2021). The effect of socio-economic status on health information literacy among urban older adults: evidence from Western China. Int J Environ Res Public Health.

[72] Zou M, Zhang Y, Zhang F (2018). The ability to obtain, appraise and understand health information among undergraduate nursing students in a medical university in Chongqing, China. Nurs Open.

[73] Harris A, Zhou Y, Liao H, Barclay L, Zeng W, Gao Y (2010). Challenges to maternal health care utilization among ethnic minority women in a resource-poor region of Sichuan Province. China Health Policy Plan.

[74] Huang Y, Martinez-Alvarez M, Shallcross D, Pi L, Tian F, Pan J, Ronsmans C (2019). Barriers to accessing maternal healthcare among ethnic minority women in Western China: a qualitative evidence synthesis. Health Policy Plan.

[75] Chen M, Qian D, Feng Z, Si L (2018). Is outpatient care benefit distribution of government healthcare subsidies equitable in rural ethnic minority areas of China? Results from cross-sectional studies in 2010 and 2013. BMJ Open.

[76] Sun W, Luo A, Bian Z (2021). Assessing the quality of online health information about breast cancer from Chinese language websites: quality assessment survey. JMIR Cancer.

[77] Wang J, Lai WF (2021). News coverage of drug development: implications for the conveyance of health information. BMC Public Health.

[78] Wang Q, Xie L, Song B, Di J, Wang L, Mo PK (2021). Using social media for health information on COVID-19 risk perceptions and mental health: an online survey of 4580 pregnant women in China. JMIR Med Inform.

[79] Wu H, Li W, Xie A, Kang L, Ke Y, Wang W (2021). Funding of health professional education: China’s 20-year process and a global comparison. Med Educ.

[80] Zhang X, Wen D, Liang J, Lei J (2017). How the public uses social media Wechat to obtain health information in China: a survey study. BMC Med Inform Decis Mak.

[81] Liuccio M, Lu J (2020). Mobile media and trust in sources of health information: a comparative study in 26 European countries. Clin Ter.

[82] Yang L, Jansz J (2021). Health information related to cardiovascular diseases broadcast on Chinese television health programs. Healthcare.

[83] Wu T, Deng Z, Zhang D, Buchanan PR, Zha D, Wang R (2018). Seeking and using intention of health information from doctors in social media: the effect of doctor-consumer interaction. Int J Med Inform.

[84] He T, Liu L, Huang J, Li G, Guo X (2021). Health knowledge about smoking, role of doctors, and self-perceived health: a cross-sectional study on smokers’ intentions to quit. Int J Environ Res Public Health.

[85] Wang X, Pan J (2016). Assessing the disparity in spatial access to hospital care in ethnic minority region in Sichuan Province, China. BMC Health Serv Res.

[86] Wang D, Li Y, Lee SG, Wang L, Fan J, Zhang G, Wu J, Ji Y, Li S (2011). Ethnic differences in body composition and obesity related risk factors: study in Chinese and white males living in China. PLoS ONE.

[87] Zhang D, Zhan W, Zheng C (2021). Online health information-seeking behaviors and skills of Chinese college students. BMC Public Health.

